# Ultrasonographic assessment of parasternal intercostal muscles during mechanical ventilation

**DOI:** 10.1186/s13613-020-00735-y

**Published:** 2020-09-07

**Authors:** Paolo Formenti, Michele Umbrello, Martin Dres, Davide Chiumello

**Affiliations:** 1 SC Anestesia e Rianimazione, Ospedale San Paolo-Polo Universitario, ASST Santi Paolo e Carlo, Milan, Italy; 2 Sorbonne Université, INSERM, UMRS1158 Neurophysiologie Respiratoire Expérimentale et Clinique, Paris, France; 3 AP-HP Sorbonne Université. Hôpital Pitié-Salpêtrière, Service de Pneumologie, Médecine Intensive-Réanimation, 75013 Paris, France; 4 Dipartimento di Scienze della Salute, Università degli Studi di Milano, Milan, Italy; 5 Centro Ricerca Coordinata di Insufficienza Respiratoria, Università degli Studi di Milano, Milan, Italy

## Abstract

Although mechanical ventilation is a lifesaving treatment, abundant evidence indicates that its prolonged use (1 week or more) promotes respiratory muscle weakness due to both contractile dysfunction and atrophy. Along with the diaphragm, the intercostal muscles are one of the most important groups of respiratory muscles. In recent years, muscular ultrasound has become a useful bedside tool for the clinician to identify patients with respiratory muscle dysfunction related to critical illness and/or invasive mechanical ventilation. Images obtained over the course of illness can document changes in muscle dimension and can be used to estimate changes in function. Recent evidence suggests the clinical usefulness of ultrasound imaging in the assessment of intercostal muscle function. In this narrative review, we summarize the current literature on ultrasound imaging of the parasternal intercostal muscles as used to assess the extent of muscle activation and muscle weakness and its potential impact during discontinuation of mechanical ventilation. In addition, we proposed a practical flowchart based on recent evidence and experience of our group that can be applied during the weaning phase. This approach integrates multiple predictive parameters of weaning success with respiratory muscle ultrasound.

## Introduction

Almost half of the patients in the intensive care unit (ICU) require mechanical ventilation (MV), and about 20% of these patients experience difficult and prolonged weaning from MV [1]. Potential contributors to ventilator-dependence include respiratory dysfunction, cardiovascular dysfunction, neuromyopathy, pharmacological influences, nutritional and psychological factors [2]. Moreover, the limited mobility of critically ill patients and disuse of their inspiratory muscles promotes the early onset of respiratory muscle dysfunction, especially when prolonged mechanical ventilation support is required (i.e., more than 1 week) [3, 4]. In the ICU, respiratory muscle function decreases dramatically, due to alterations in contractile properties that decrease in fatigue resistance indexes [5, 6] and atrophy [7]. Although it is likely that the diaphragm is much more sensitive than any other muscle to disuse, intercostal muscles too, along with other major respiratory muscles, are thought to be involved [8, 9].

Many tools are available to monitor and evaluate respiratory muscle function [10, 11]. Among these, the most extensively studied have traditionally been those which evaluate the diaphragm function [12–14]. Current knowledge about the intercostal muscle function primarily has been based on the results of a relatively small number of electromyographic studies relating to chest wall shape and motion [15–17] or to indices of inspiratory effort detected by electromyographic activity [18]. Although providing useful data about muscular activation, this methodology is seldom used in clinical practice and, most importantly, does not provide information about muscle dimensions, shape, and motion. Moreover, it may be prone to low-quality recordings influenced by noise and artifacts, and requires off-line analysis performed by a specialist to be interpreted [19]. In contrast, ultrasound may represent a potential modality to integrate these evaluations, assessing muscular activity and structure also in diverse respiratory muscle groups. In particular, ultrasound-guided estimation of muscle dimension (by measurement of muscle thickness) and the ultrasound-guided estimation of weakness (by the assessment of muscle contraction during spontaneous or assisted breathing) has recently been advocated as a simple and reliable method for such a bedside evaluation [20–22]. Similar to the diaphragm, monitoring the function of parasternal intercostal muscles might help to identify and quantify the degree of activation of inspiratory muscles during assisted breathing. Unlike other extra diaphragmatic inspiratory muscles of the neck (such as sternocleidomastoids or scalenes) which are increasingly recruited during voluntary inspiratory tasks [23], the ultrasound window of the parasternal intercostals is almost always accessible in the supine position. Accurate muscle measurement depends not only on operator skills (e.g., measurement location, probe angulation), but also on technical aspects related to ultrasound physics and patient characteristics. While in general ultrasound seems to be a reliable technique, comparing individual patient results must be done with a degree of caution and only after adequate training, as small observer-dependent variations will affect results [24–26]. The aim of this review is to define the role of the intercostal muscles and their ultrasound evaluation during MV. To identify relevant papers, a bibliographic search was conducted accessing the following databases: PubMed, Cochrane Library, Scopus, Web of Science, from inception to the cutoff date of February 29th, 2020. To supplement the search, the reference list of every paper was also manually screened to identify additional potentially eligible studies. The following key-words were used, alone or combined with appropriate Boolean operators, to search the different databases: “intercostal muscle”, “extra diaphragmatic muscle”, “mechanical ventilation weaning”, “respiratory muscle ultrasound”, “intercostal muscle ultrasound”, “parasternal intercostal muscle ultrasound”, “critically ill patients”, “critical illness”, “intensive care”. A similar search was also performed using the PubMed MeSH thesaurus. The final search identified 89 eligible studies, which were subsequently and independently screened and underwent full-text review to identify 21 studies.

### Anatomy and physiology of respiratory muscles

**Fig. 1:**
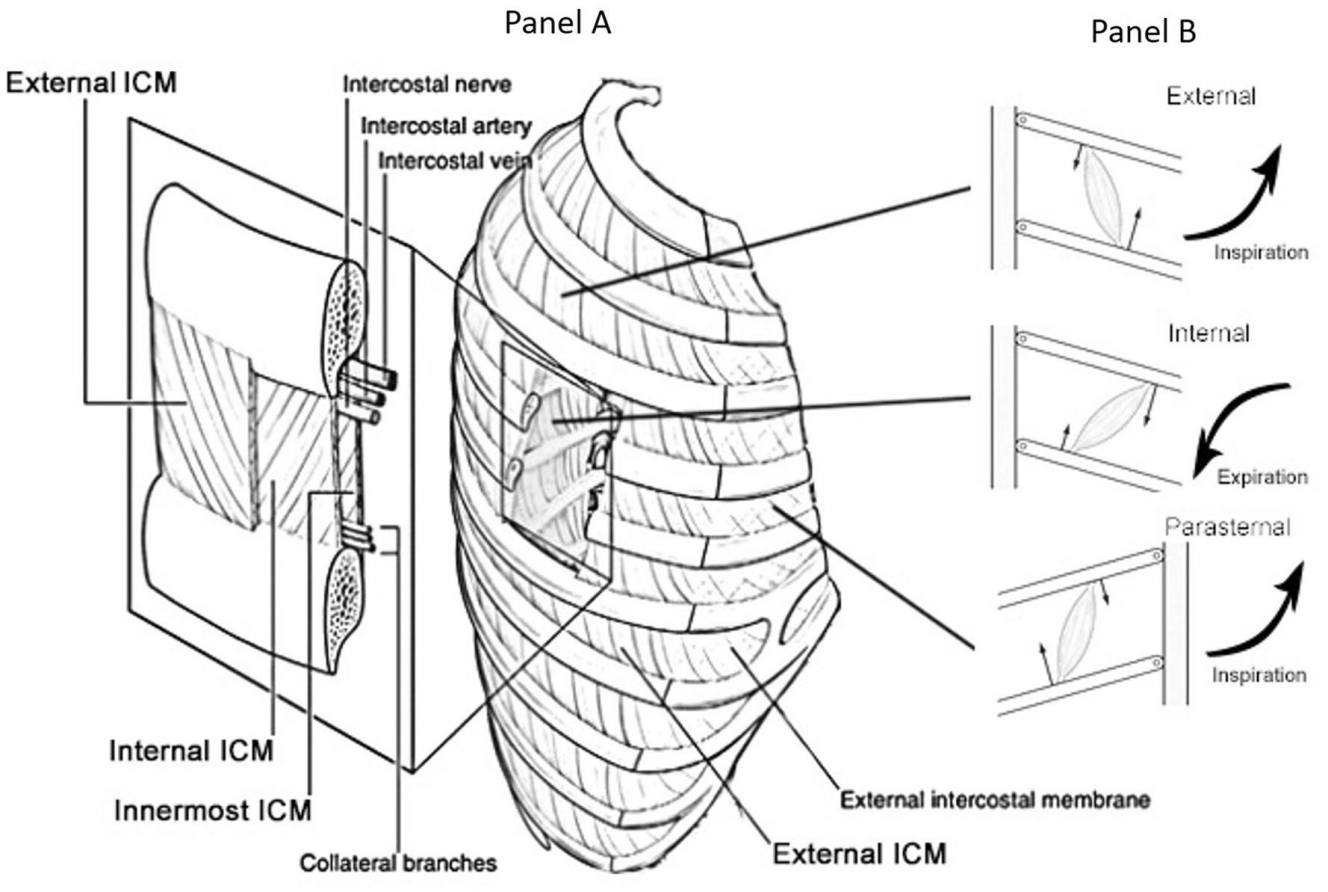
Intercostal muscles anatomy. A schematic picture of anatomy and function of internal and external intercostal muscles. **Panel A**: the figure represents the anatomical distribution of intercostal muscles.** Panel B**: the figure represents the schematic functional activity of intercostal muscles. In particular, the external intercostal connects the ribs in such a way that contraction of the muscles lift the ribs and the rib cage allowing to expand the anterior–posterior dimension of the chest wall. On the other hand, the contraction of the internal intercostal (oriented opposite to the external) determines the opposite effect such as lowering of the ribs and reducing the anterior–posterior dimension

Together with the diaphragm, extra-diaphragmatic inspiratory muscles participate in the generation of the tidal volume. The intercostal muscles are composed of three thin layers of muscle fibers occupying each of the intercostal spaces. The external intercostals extend from the tubercles of the ribs dorsally to the costochondral junctions ventrally, and their fibers are oriented obliquely, downward, and forward, from the rib above to the rib below (Fig. 1, Panel A). Ventrally, between the sternum and the costochondral junctions, the fibers are those of the internal intercostal muscles; these are particularly thick in parasternal region of the rib cage, where they are conventionally called the parasternal intercostals. These anatomical structures are active during the inspiratory phase and interact with the diaphragm and other extra diaphragmatic inspiratory muscles [27]. In contrast, the internal intercostal muscles are directed obliquely, upward, and forward from the superior border of the rib and costal cartilage below to the base of the subcostal border of the rib. The innermost intercostal muscles are the deepest, separated from the internal intercostal muscles by a grouping of nerves and blood vessels known as the neurovascular bundle [15]. These internal intercostals are active during the expiration phase of respiratory cycle (Fig. 1b). The complex interactions among all these fiber groups have been investigated in few physiological humans’ studies. The inspiratory drive to the external intercostal muscles was assessed by De Troyer et al. [8], who showed how the third dorsal external intercostal was active earlier in inspiration. Moreover, the degree of activation of the external intercostal muscles during quiet breathing correlated with the magnitude of their mechanical advantage, which diminishes fourfold from the second to the fifth interspace [17]. The same authors, in a complex physiological study, showed how the parasternal intercostals have an inspiratory action on the lung even if with a pressure-generating ability less remarkable as compared to other extra diaphragmatic muscles [16]. Moreover, the study described how the fractional shortening of the parasternal intercostals decreased gradually from the second to the fifth interspace. Nevertheless, it remains unknown whether the neural drive to parasternal intercostal muscles is graded across the interspaces [28]. The spatial distribution of neural drive along rostro-caudal gradient has been demonstrated in a study in which the timing of activation and the discharge performance of single motor units activated during inspiration in the parasternal intercostals of the first to fifth interspaces were measured [29]. During respiratory loading, Sampson et al. [30] showed how the pattern of chest wall deformation was closely related to the activity and coordination of the various inspiratory intercostal muscles, and how the parasternal intercostals do not necessarily represent all inspiratory intercostals. In summary, the movement of the ribs depends on the relative amount of torque around the vertebral articulations acting on the two points of attachment of the muscle to the respective ribs: when external intercostal muscles contract, the torque acting on the lower rib is greater than that acting on the upper rib, with a net effect to raise the ribs. The reverse actually happens for the internal intercostals, which act to lower the ribs to which they attach. Eventually, the parasternal intercostals raise the ribs as their action is transmitted to the sternum (Fig. 1b).

### Ultrasound measurements of intercostal muscle

Ultrasound imaging has been advocated as an interesting imaging modality as it has been shown to be accurate, reproducible and convenient tool for the assessment of linear dimensions, cross-sectional area, thickness, and indices of muscle architecture [31, 32]. In addition, ultrasound has already been used to assess the relationship between muscles and chest wall motion [33, 34]. An animal model demonstrated the ability of ultrasound to accurately estimate the cross-sectional area of the parasternal intercostal muscles, comparing it with the measurement directly performed on the specimen [35]. In healthy volunteers, ultrasound has been used to investigate parasternal intercostal muscles in the easily accessible anterior parasternal region [35, 36]. Conversely, in the lateral and in the posterior part of the intercostal space, the internal and external intercostal muscles often overlap, making the ultrasound detection of both muscle layers impossible. Therefore, the increasing muscle thickness of the external intercostal muscle during inspiration (an estimate of muscle recruitment) cannot be assessed in this location.

Using ultrasound imaging in healthy volunteers, Cala et al. [33] reported that the muscles move ventrally and straighten. Cala et al. [36] measured the inter-rib distance, i.e., the parasternal intercostal muscle thickness. The authors showed a ventral movement during tidal breathing without changes in inter-rib distance and thickness, thereby concluding that the parasternal intercostal muscle moves ventrally and more perpendicular to the ribs. The findings support an intercostal stabilizing function of the parasternal intercostal muscles. Wallbridge [24] postulated that the relationship between intercostal muscle sizes might reflect different roles and recruitment that depends on chest wall site. This hypothesis was based on weak correlation (*r* = 0.33) observed between ultrasound thickness of parasternal intercostals and severity of airflow obstruction as measured by spirometric parameters (i.e., FEV1% predicted) in stable COPD patients. Given the preliminary nature and the weak correlation of the results, this topic deserves to be explored more thoroughly. A recent study by Yoshida et al. [26] used ultrasound to assess whether human parasternal intercostal muscle thickness increased during vigorous breathing efforts (the maximal inspiratory level using an inspiratory resistance loading device). The images obtained showed an increase in muscle thickness only in the anterior portion of the intercostal space during maximal breathing, whereas no significant differences could be detected in the lateral and posterior portions. This remark suggest how the parasternal intercostal portion may be the best region to investigate the intercostal muscle, as in the lateral and posterior part of the intercostal space, internal intercostal muscles and external intercostal muscles overlap, and ultrasound cannot separate both muscle layers. Nevertheless, these observations must be investigated more thoroughly in mechanically ventilated patients. Regarding the reproducibility of ultrasound technique, a high interobserver reproducibility of parasternal intercostal end-expiratory and peak-inspiratory thickness has recently been confirmed [37] as previously reported [35, 36]. Moreover, intra-rater reliability of intercostal muscle thickness depended on site, ranging between 0.87 and 0.97 [24]. Relevant observations are summarized in Table 1.

**Table 1:** Principal studies regarding intercostal muscle ultrasound

Study and year of publication	Design	Setting of the study	Main remarks
Cala et al. 1998 [33]	Four healthy subjects	7.5-MHz curvilinear phased array transducer to obtain ultrasound image of the second right and left interspace in the sagittal plane during tidal breathing and at residual volume, functional residual capacity, and total lung capacity. Inter-rib distance, parasternal intercostal thickness, and motion of the midpoint were measured	During inspiration, the parasternal intercostal muscle moves ventrally and straightens, and lung volume influences its shape and motion. The findings support an intercostal stabilizing function of these muscles
Diab et al. 1998 [31]	Experimental and one healthy volunteer trial	Measurements of the area of the intercostal muscles in normal man during maximal inhalation and exhalation. The 5-MHz probe was placed perpendicular to the chest wall and parallel to the longitudinal axis of the body. The probe was passed over a line at the medial border of the left and right scapulae. Measurements started in an upward direction from the intercostal spaces between the 11th and 12th ribs to those between the 5th and 6th ribs	There is symmetrical expansion of the intercostal muscle area and indirectly of muscular activity at maximal inhalation and maximal exhalation. This finding emphasizes the role of the intercostal muscles in keeping the chest wall in balance during breathing. Measurements of the intercostal muscle area between the 5th and 12th ribs approximately at the medial scapular line computed from the perpendiculars of the area at maximal inhalation give more reliable results
Yoshida et al. 2019 [35]	Twelve healthy subjects	Intercostal muscle thickness measured using ultrasound at rest and at maximal breathing in the anterior, lateral, and posterior parts of the right intercostal spaces	The thickness of the intercostal muscle showed significant increases in the first, second, third, fourth, and sixth intercostal spaces of the anterior portions. There were no significant differences in the lateral or posterior portions between rest and maximal breathing
Nakanishi et al. 2019 [47]	80 ICU MV patients	Ultrasound intercostal muscle thickness was measured on days 1, 3, 5, and 7	Intercostal muscle thickness was associated with prolonged mechanical ventilation and length of ICU stay
Wallbridge et al. 2019 [34]	20 COPD patients	Ultrasound measurement of thickness and echogenicity of 2nd and 3rd parasternal intercostal muscles, dominant pectoralis major and quadriceps, and diaphragm thickness; spirometry; and chest CT	Ultrasound intercostal thickness moderately correlated with FEV1% predicted and quadriceps thickness. Echogenicity correlated negatively with FEV1% predicted; CT-measured lateral intercostal mass correlate negatively with parasternal ultrasound intercostal thickness. Changes in muscle quantity and quality reflected spirometric disease severity
Dres et al. 2020 [36]	Twenty healthy subjects; 16 patients in PSV. Fifty-four patients during SBT	Ultrasound of parasternal intercostal muscle at the level of 2nd right intercostal space; thickness of the parasternal intercostal muscle measured at end expiration and at peak inspiration. Change in thickness determined the thickening fraction of the parasternal intercostal muscle	There was a progressive decrease in parasternal muscle thickening fraction with increasing levels of PS and an inverse correlation between parasternal muscle thickening fraction and the pressure generating capacity of the diaphragm. The parasternal muscle thickening fraction was higher (17%) in patients with diaphragm. The pressure generating capacity of the diaphragm, the diaphragm thickening fraction and the parasternal thickening fraction similarly predicted failure or the spontaneous breathing trial
Umbrello et al. 2020 [38]	Twenty-one ICU MV patients in PSV	Parasternal intercostal ultrasound (thickness and thickening fraction) at three-level of PS (baseline, 25% and 50% reduction). Concomitant recording of the esophageal and transdiaphragmatic pressure–time products, work of breathing, and diaphragm ultrasound	Additional measurement of parasternal intercostal thickening may discriminate a low inspiratory effort or a high effort in the presence of a dysfunctional diaphragm

### Ultrasound features and settings

**Fig. 2:**
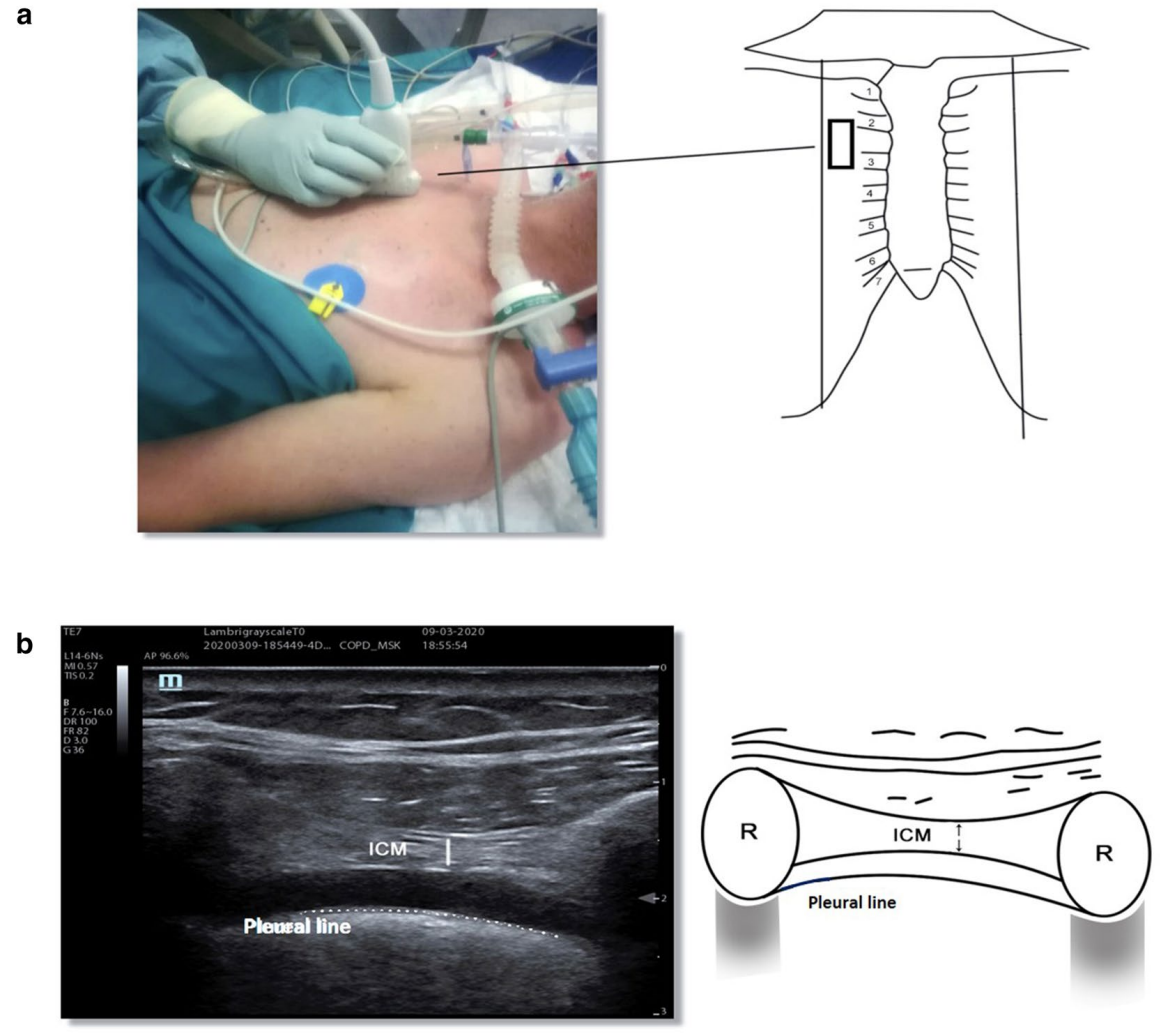
Intercostal muscle ultrasound. The figure depicts an ultrasound image of parasternal intercostal muscle ultrasound. **a** The ultrasound probe position at the parasternal space. **b** The ultrasound image depicted by ultrasound using a B-mode setting in which the intercostal muscle lies between two ribs (R), cranial to the pleural line, behind the pectoral muscle. The left side is a schematic view of the ultrasound image. *ICM* intercostal muscle

The parasternal intercostal muscles can be assessed using a 10–15 MHz, linear ultrasonography transducer in M-mode, placed 3–5 cm laterally from the sternum, and oriented transversally in the sagittal plane between the 2nd and the 3rd rib [37, 38]. For instance, while the patient is lying at a 20° head-up position, the linear array transducer is positioned perpendicular to the anterior thorax surface in the longitudinal scan. Starting in B mode, the pleural line is easily detected as a part of the “bat sign” [39]. Just above the pleural line, the parasternal intercostal muscle can be identified as a three-layered biconcave structure, where two linear hyperechoic membranes running from the anterior and posterior aspects of the adjoining ribs, and a medial portion with muscle echotexture (Fig. 2) [24, 37]. Muscle thickness is measured between the inner and outermost hyperechogenic layer of the muscle fascial borders. Moreover, using the M-mode, the inspiratory contraction of the muscle can be detected, as described for the thickening fraction of the diaphragm [40]. In fact, during the inspiration phase, an increase in muscle thickness can be visualized as the muscle fiber contraction displaces the rib cage cranially and anteriorly, while muscle mass is maintained constant [27]. Parasternal intercostal muscle-thickening fraction (TFic) can be calculated as the ratio of the difference between end-expiratory and end-inspiratory thickness to end-expiratory thickness (TF = ((TH end inspiration – TH end expiration)/TH end expiration)) * 100), similarly to the diaphragm thickening fraction (TFdi). Furthermore, indirect qualitative information on muscle composition/structure can be recorded by measuring muscle echogenicity (image-grey-scale) [41]. Muscle echogenicity can define the region of interest for analysis performed using a standard histogram function widely available in many commercial software for image editing [42]. Higher muscle echogenicity (indicating poorer muscle quality) of parasternal intercostal muscle is negatively correlated with COPD severity [24]. However, since echogenicity measurements are influenced by observer-dependent factors more strongly than are other measures, a rigorous ultrasound setting must be selected before acquiring the image to standardize the methods. Some features regarding the probe orientation must also be considered when exploring parasternal muscles. In fact, it has been supposed that the parasternal intercostal muscle image quality progressively reduces as the angle of incidence of ultrasound changes by moving the transducer away from the parasagittal plane. In a pioneering study, Wait et al. [43] showed how the value of the misalignment angle—i.e., the angle at which the transducer no longer produces a readable signal—ranged from 1.6° to 8.4° for transducer frequencies between 10 and 1 MHz, respectively, during the evaluation of diaphragm movements. Such observations have never been tested for the evaluation of intercostal muscle. Since the ventral and dorsal edges of the parasternal intercostal muscles are not parallel in the majority of the images, the thickness measurements should be performed at the cranio-caudal midpoint between the ribs. There, the difference in the curvature between the two surfaces is least, and the lines flattest.

### Parasternal intercostal muscle changes during mechanical ventilation

Even if only few studies have focused specifically on the intercostal muscles in mechanically ventilated patients, some recent papers investigated the effects of MV on extra diaphragmatic respiratory muscles (Table 1). Starting from animal observations, Capdevilla et al. [44] showed how MV in rabbits produces alterations in contractile properties of the 5th external intercostal muscle, increasing muscle fatigue and promoting atrophy of type II fibers. More specifically, Bernard et al. [45] showed a disruption of myofibrils in both the diaphragm and the 5th and 6th external intercostal muscles.

Direct extrapolation of these scarce results to human subjects may be questioned; however, a time-dependent relationship between the duration of ventilation and the subsequent development of weakness appears likely [37, 46, 47]. In this regard, whereas the effects of MV on the diaphragm have extensively been studied [10, 22], there is little evidence regarding its effects on the performance of other inspiratory muscles and the relative contribution of intercostal muscle dysfunction remains to be determined, especially in ventilator-dependent patients. Nakanishi et al. [48] showed how an excessive inspiratory support causes atrophy of the disused diaphragm and intercostals muscle and that excessive pressure might cause injury to respiratory muscles and structural changes with gradually increasing thickness. Recently, Dres [37] reported that the TFic was significantly associated with failure of a spontaneous breathing trial in mechanically ventilated patients and how this thickening was responsive to the level of ventilator assistance and significantly higher in patients with diaphragm dysfunction. More specifically, a TFic exceeding 8% identified patients with diaphragm dysfunction, and a value greater than 10% predicted weaning failure. In support of this observation, Umbrello et al. [38] showed similar results, describing a complex relationship between the diaphragm and parasternal intercostal muscles. The authors showed how diaphragm thickening fraction was higher (> 30%) and that of parasternal intercostal was lower (< 5%) in patients without as compared to patients with diaphragm dysfunction. In the presence of diaphragm dysfunction a low TFdi may reflect a low inspiratory effort, or an elevated effort with inspiratory work performed by extra-diaphragmatic muscles associated with low or elevated levels of Tfic, depending on level the mechanical respiratory support delivered by the ventilator. While we acknowledge that these are recent and preliminary observations, and more importantly given the monocentric and pilot nature of the studies, their consistency and strong physiological rationale led us to propose their use in clinical practice.

### Practical approach and potential applications

**Fig. 3:**
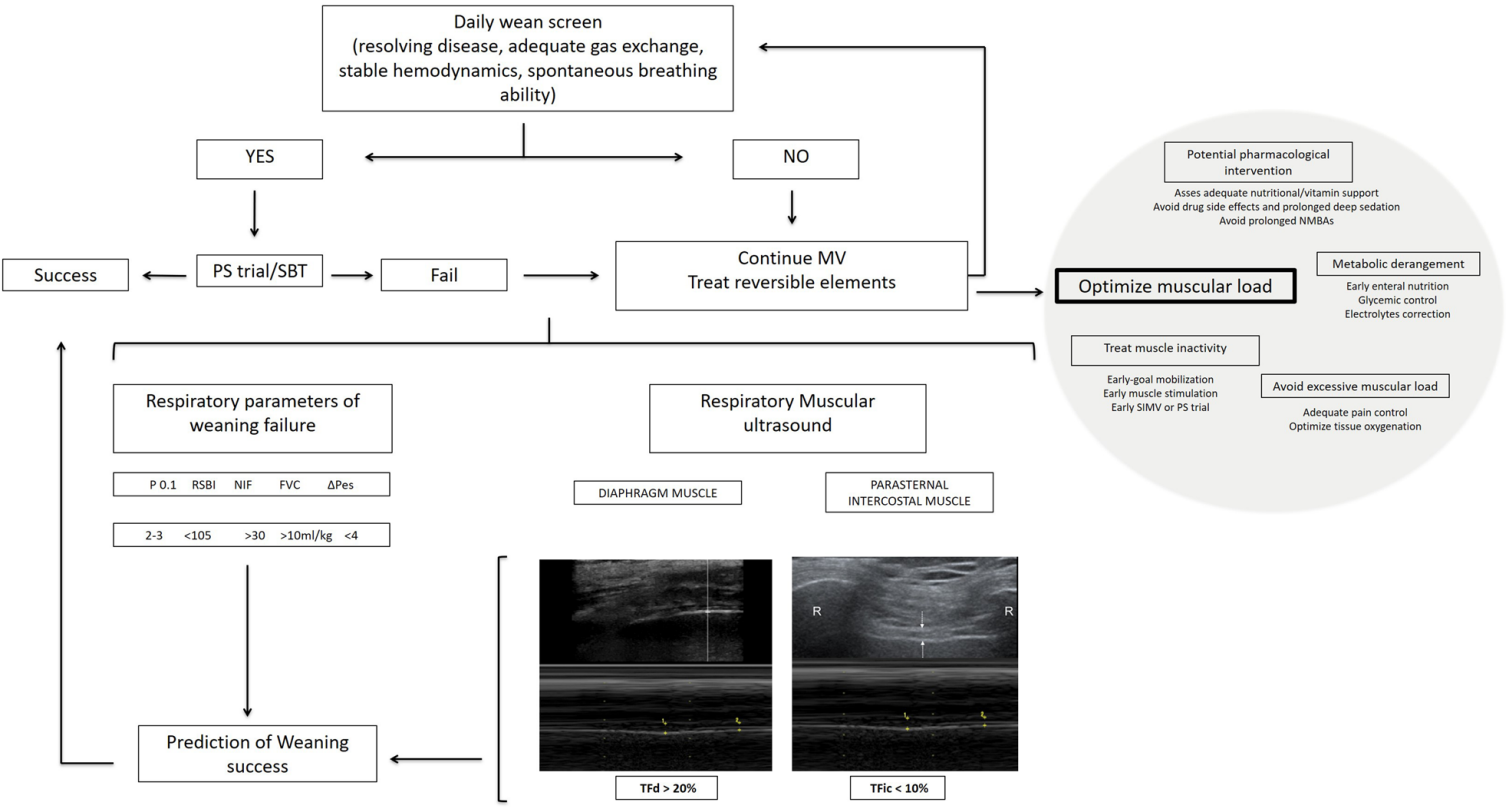
A simplified diagram of respiratory muscular ultrasound during weaning trial from mechanical ventilation. Once a pressure support (PS) trial or a spontaneous breathing trial (SBT) is started and patients failed a termination of the weaning process within 24 h a diaphragm evaluation exploring the diaphragmatic thickening fraction (TFdi) should be performed. At the same time, the parasternal intercostal muscle should be evaluated. The simplest method is to evaluate the thickness and the thickening fraction (TFic) as calculated for the diaphragm (TF = ((TH end inspiration – TH end expiration)/TH end expiration)) * 100. A value of TFic less than 10%, associated with a TFdi greater than 20% indicates a pattern of breathing in which respiratory muscles are not recruited, and is then suggestive of a successful weaning trial. All these measurements should be integrated with other routinely used predictive parameters of weaning failure, such as rapid shallow breathing index (RSBI), negative inspiratory function (NIF), forced vital capacity (VFC) and the tidal change in esophageal pressure (Δpes). In any case, consider optimizing muscular load acting on pharmacological intervention, metabolic supply, and respiratory muscular in/activity

Based on the recent consistent findings on this topic [37, 49], we propose a practical flowchart during the weaning trial from MV, in which ultrasound muscular evaluation of the whole respiratory apparatus is taken into account. A similar approach has recently been suggested in an ABCD ultrasound flowchart [49]. Weaning failure has a multifactorial nature, which results from muscular dysfunction, excessive mechanical load, weaning-induced cardiovascular dysfunction, or reduced ability to clear airway secretions. Most physicians simply evaluate the patient’s ability to tolerate a spontaneous breathing trial (SBT) without distress to determine likely weaning success [50, 51]. We propose that once a SBT is started, a diaphragm evaluation exploring the TFdi should be considered in those patients who failed a termination of the weaning process within 24 h [1]. At the same time, we propose to evaluate the parasternal intercostal muscles (Fig. 3). This suggestion derives from considering that even when low contractile activity of the diaphragm is identified, two different scenarios may be present: the diaphragm evaluation may be in the normal range because of an overassisted machine setting, or this reduced activity may signify diaphragm dysfunction. In the latter case, the involvement of parasternal intercostals should be considered, as a significant thickening of these muscles may be a sign of muscle recruitment. Thus, a value of TFic less than 10%, associated with a TFdi greater than 20% is suggestive of a breath in which the diaphragm is the main inspiratory muscle and the extra diaphragmatic muscle is not “stressed”, thereby potentially leading to a successful weaning trial [37, 38]. Otherwise, it may be possible that, in the presence of diaphragm dysfunction, the intercostal muscles have become more active, which is a negative indicator, potentially predictive of weaning failure. The clinicians must consider that these thresholds have not been prospectively validated, and that all these measurements may be integrated with other commonly used predictive parameters of weaning success, such as the rapid shallow breathing index (RSBI), the negative inspiratory pressure (NIP), forced vital capacity (FVC) [52], gas exchange and the tidal changes in esophageal pressure (Δpes) [53]. These, however, are not always investigated, and each is characterized by its own specificity and sensitivity. While we are well aware that it is simplistic to think that a single parameter could predict overall weaning success or failure, we nevertheless think that the strong physiological rationale of parasternal intercostal ultrasound may be of help to the clinician after an adequate training. With the exception of chronic muscular disease [54], the contributions to respiratory failure from respiratory muscles other than the diaphragm, and the possible causative role of prior muscle inactivity, still are poorly understood. No study has specifically investigated the role of intercostal muscles during support by different respiratory devices, such as non-invasive ventilation.

## Conclusions

The available evidence suggests a mechanism by which the parasternal intercostals, and potentially other inspiratory muscles, may contribute to the inspiratory fall in pleural pressure by preventing paradoxical inward displacement of the upper rib cage and decreasing ribcage distortability. Moreover, unphysiological contraction (i.e., thickening) of such muscle groups may be the sign of an excessive recruitment of accessory muscles, such as in the case of underassisted breathing. This may be investigated by using the ultrasound during weaning from MV. Muscle architecture and its complex interrelationship with the muscular and bony rib-cage apparatus require further careful investigation. The intriguing but preliminary nature of the available data highlights the need for more detailed models of intercostal mechanics than presently available to assist our understanding of this complex area of respiratory mechanics during MV.

## Data Availability

Not applicable.

## Abbreviations

ICU: intensive care unit
MV: mechanical ventilation
DD: diaphragmatic dysfunction
TFic: intercostal thickening fraction
TFd: diaphragmatic thickening fraction
SBT: spontaneous breathing trial
NIP: negative inspiratory pressure
RSBI: rapid shallow breathing index
Δpes: tidal changes in esophageal pressure
FVC: forced vital capacity
